# Automated Image Analysis in the Evaluation of Facial Pigmentation in Melasma: Clinical Application of Pycnogenol

**DOI:** 10.1111/jocd.71274

**Published:** 2026-11-05

**Authors:** Sullege Fabiola Suzuki, Carlo Ralph de Musis, Walkiria Shimoya‐Bittencourt

**Affiliations:** ^1^ University of Cuiabá (UNIC) Cuiabá Brazil; ^2^ Pontifical Catholic University of São Paulo (PUC‐SP) São Paulo Brazil; ^3^ Federal University of São Paulo (UNIFESP) São Paulo Brazil

**Keywords:** artificial intelligence, melasma, pigmentation, pycnogenol

## Abstract

**Introduction:**

Melasma is characterized by chronic hyperpigmentation and has a significant psychosocial impact. Traditional clinical assessments are limited due to subjectivity and low sensitivity to detect small changes.

**Objective:**

To evaluate the progression of facial pigmentation in patients undergoing melasma treatment over 60 days using automated image processing based on artificial intelligence.

**Methods:**

This prospective clinical trial enrolled 30 individuals with facial melasma into intervention (Pycnogenol 75 mg/day) and control group. Standardized photographs were obtained at baseline (T_0_), 30 days (T_30_), and 60 days (T_60_). Images were processed using a computational pipeline that included automatic renaming, facial segmentation with MediaPipe, extraction of the mean intensity across RGB channels, and application of principal component analysis. Linear mixed‐effects models assessed the effects of group, time, and group‐by‐time interaction.

**Results:**

PC1 accounted for 99.35% of the variance in gray intensity, indicating a robust marker of pigmentation. A significant effect of time was observed (*p* = 0.048), indicating overall changes in pigmentation over the 60‐day period, with greater changes during the first 30 days. Group‐by‐time interaction showed *p* = 0.056, suggesting a trend toward greater response in the intervention group. This group was also marked by reduced PC1 at T_30_, followed by stabilization at T_60_.

**Conclusion:**

The automated analysis of facial images proved to be an objective tool for monitoring melasma and detecting subtle changes not captured by subjective clinical methods. Treatment with Pycnogenol showed greater lightening than placebo, particularly during the first month.

## 1 Introduction

Melasma is a chronic pigmentary disorder characterized by irregular, persistent hyperpigmentation predominantly in sun‐exposed areas of the face of women aged 25 to 50 years and individuals with intermediate to high phototypes. Although it does not pose a direct health risk, melasma has a significant psychosocial impact, affecting self‐esteem, social relationships, and quality of life [1].

The pathophysiology of melasma is multifactorial and involves genetic predisposition, hormonal stimuli, ultraviolet radiation, visible light, dermal inflammation, and oxidative stress [2]. Among these mechanisms, oxidative stress has been identified as a key contributor to the persistence of hyperpigmentation, promoting melanocyte hyperactivity, inflammatory dysregulation, and sustained skin damage [3].

Although recent therapeutic advances have improved the management of melasma, assessing treatment response remains a methodological challenge. Traditional clinical instruments, such as the Melasma Area and Severity Index, are limited by their subjective nature, dependence on evaluator experience, and interobserver variability [4]. Even semiquantitative methods may fail to detect small but clinically relevant changes in pigmentation.

A promising alternative involves approaches based on machine learning, computer vision, and quantitative image analysis. These methods convert color information (RGB) into reproducible objective numerical metrics that are sensitive to small changes in pigmentation. Robust algorithms (e.g., automated facial segmentation, principal component analysis [PCA], and repeated‐measures statistical models) also provide data that quantify pigment changes with greater precision than clinical scales [5, 6].

Automated image processing pipelines offer several advantages, including elimination of subjective bias, increased sensitivity to small variations, reproducibility across evaluators and centers, and standardized assessment of pigmentation over time. The incorporation of such tools represents an important methodological advance in aesthetic and cosmetic dermatology, particularly for conditions in which clinical progression involves subtle and gradual changes [7].

Therefore, this study aimed to evaluate changes in facial melasma pigmentation over 60 days using an objective method based on automated image analysis, including facial segmentation, extraction of RGB intensities, data consolidation via PCA, and statistical modeling with linear mixed‐effects models (LMM). This approach provides a quantitative and highly sensitive marker of treatment response and supports the advancement of instrumental methods for melasma assessment.

## 2 Materials e Methods

### 2.1 Study Design

This prospective clinical trial was approved by the research ethics committee of the University of Cuiabá (UNIC) (No. 7.042.457) and conducted at the Functional Research Laboratory at the UNIC. The protocol was initiated after ethical approval and written informed consent from all participants.

### 2.2 Population and Eligibility Criteria

A total of 30 participants of both sexes, aged 18 to 59 years, with a clinical diagnosis of facial melasma were included. Inclusion criteria were clinical diagnosis of melasma, age between 18 and 59 years, no topical treatment or procedures for melasma in the previous six months, and availability for a 60‐day follow‐up.

Those pregnant, breastfeeding, using oral contraceptives, hormone therapy not compatible with the protocol, anabolic agents, antioxidants from other classes, or medications or metabolites associated with hyperpigmentation were excluded. Participants with clinical conditions that could interfere with safety or study outcomes were also excluded.

### 2.3 Clinical and Demographic Data

Clinical and demographic data were recorded using a standardized data collection form that included coded names, age, weight (kg), height (meters), history of skin disease, current medications, use of contraceptives or hormone therapy, age at onset of lesions, lesion progression, previous treatments, and adherence to sunscreen use. Although the primary outcome was objective image analysis, these data were collected to ensure full sample characterization.

### 2.4 Image Standardization

Images were obtained using the Observ 520× (Sylton Diagnostic Systems, Son, The Netherlands) under strictly standardized conditions to ensure reproducibility and minimize variability across time points. All photographs were captured in a controlled environment, with fixed ambient lighting and no external light interference, using the device's built‐in illumination system and standardized optical filters (e.g., cross‐polarized and UV modes), applied in the same sequence for all participants. Individuals were positioned at a fixed distance using the device's positioning support, with head alignment standardized by anatomical references, maintaining a neutral facial expression, closed mouth, and relaxed musculature. Images were acquired at predefined angles (frontal and lateral views) following the same protocol at baseline (T0), 30 days (T30), and 60 days (T60). All acquisitions were performed using identical camera settings (resolution, focus, and exposure), without manual adjustments between sessions. Participants were instructed not to use makeup, sunscreen, or topical products prior to image capture, and all images were exported in high resolution for subsequent computational analysis.

### 2.5 Image Processing Pipeline

Image processing was performed using a computational pipeline consisting of five sequential steps developed in Python. Initially, the images were automatically organized and renamed through a recursive scan of the original directories, from which the patient identifier, capture angle, and applied lighting filter were extracted and consolidated into a standardized naming convention. Subsequently, automatic facial segmentation was performed using the FaceMesh model from the MediaPipe library, which detects 468 facial landmarks per image and enables the creation of a binary mask based on the convex hull of these points, from which the eye, eyebrow, and mouth regions are subtracted; the remainder of the image is filled with pure green (0,255,0). In cases where the facial mesh was not detected, an auxiliary bounding‐box‐based method (FaceDetection) was applied. This segmentation isolates the facial skin area, over which pigmentation is quantified globally (Figure 1). The processed images were saved with prefixes indicating successful or failed segmentation.

**FIGURE 1:**
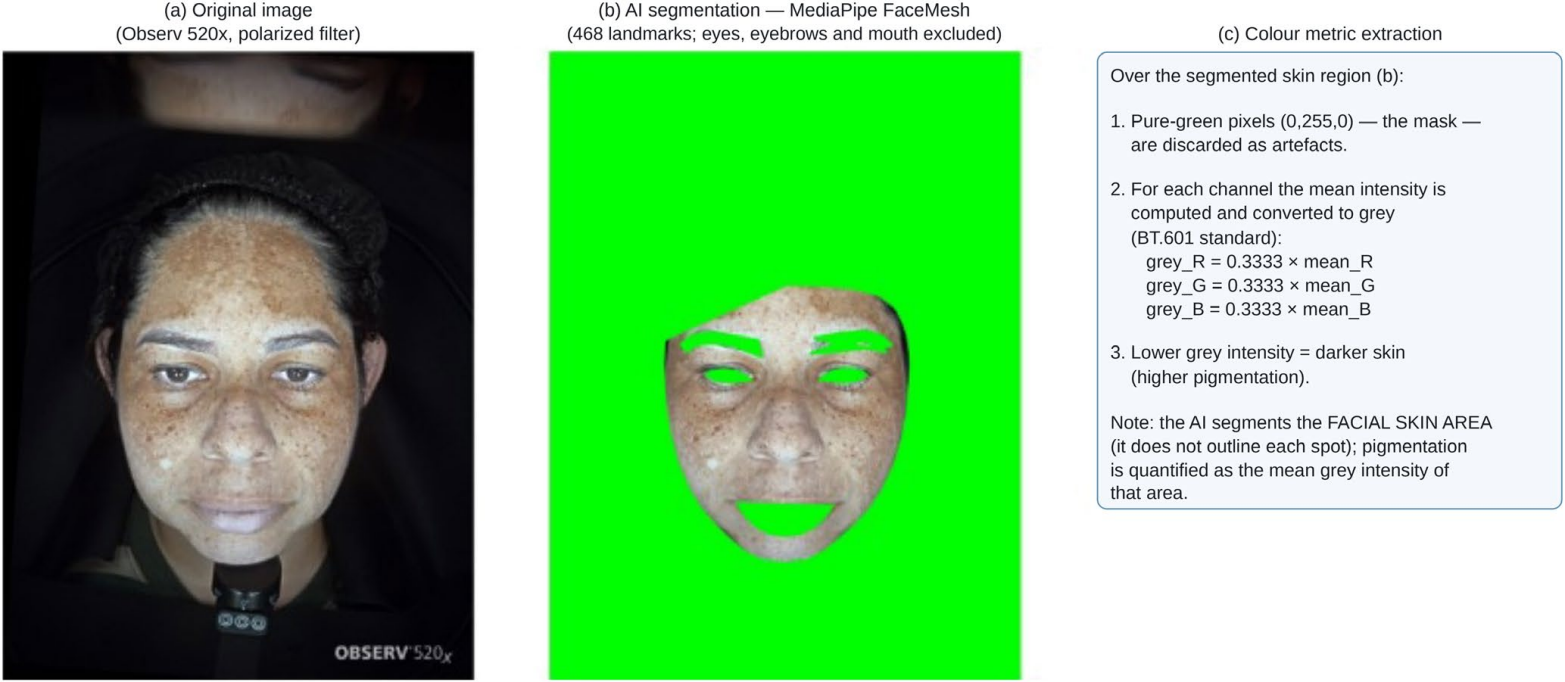
Automated facial segmentation using artificial intelligence (MediaPipe FaceMesh): (a) original image; (b) segmentation of the skin area with exclusion of the eyes, eyebrows, and mouth; (c) extraction of mean color metrics by channel (BT.601 standard).

Subsequently, each segmented image was analyzed for RGB color composition. Pure green pixels (0, 255, 0) were treated as artifacts and excluded, and the mean gray intensity for each channel was calculated according to the BT.601 standard. Last, a standardized temporal variable (in days) was created by identifying the baseline date (T_0_) for each patient and calculating the number of days elapsed for each subsequent image. The output of this pipeline was a final dataset for statistical analysis.

### 2.6 Duration and Evaluation Schedule

Interventions and evaluations were conducted at baseline (T_0_), 30 days (T_30_), and 60 days (T_60_). Clinical evaluation and data collection were performed at T_0_, while melasma assessments (standardized images) were conducted at T_0_, T_30_, and T_60_. Laboratory tests (complete blood count and liver function tests) were collected at T_0_ and T_60_ to monitor hematological and hepatic safety.

### 2.7 Interventions

Individuals were divided into intervention and control groups. The former received oral pycnogenol once daily at a dose of 75 mg, which is within the commonly reported therapeutic range (50 to 100 mg/day). The control group received a placebo tablet (no active substance), administered orally once daily. Both groups were instructed to use SPF 30 sunscreen every day throughout the study, and sunscreen was provided to all participants.

### 2.8 Primary Outcome

The primary outcome analyzed was the change in facial pigmentation, assessed by transforming the gray_R, gray_G, and gray_B metrics using principal component analysis (PCA), in which the first principal component (PC1) accounted for most of the variance among the original variables. This component was used as an objective pigmentation index and interpreted as an intensity marker, with higher values indicating greater skin darkening, whereas progressive reductions were interpreted as skin lightening over time.

### 2.9 Statistical Analysis

Data were shown as estimated marginal means and standard error. Normality and homoscedasticity of residuals were assessed using the Shapiro–Wilk and Levene tests. LMM were used to assess the effects of group (intervention and control), time (T_0_, T_30_, and T_60_), and group × time interaction on PC1, including a random intercept for each participant to account for repeated measures. Estimated marginal means at T_0_, T_30_, and T_60_ were compared using Tukey's post hoc test, and *p* < 0.05 was considered statistically significant. Statistical analysis was performed using the R software (version 4.3.2).

## 3 Results

### 3.1 Sample Characteristics

A total of 30 participants (90% females; mean age of 38.2 ± 9.1 years; Fitzpatrick skin phototypes III and IV) with facial melasma were assigned equally to the intervention (pycnogenol, *n* = 15) and control (placebo, *n* = 15) groups. Individuals completed all evaluations up to T_60_.

All participants maintained the regular use of sunscreen, and no severe adverse reactions to treatment were reported. Laboratory tests (complete blood count and liver function tests) remained within normal ranges at T_60_, with no clinically relevant changes.

### 3.2 Pigmentation Analysis Using PCA

Principal component analysis (PCA) applied to the gray_R, gray_G, and gray_B variables demonstrated that PC1 explained 99.35% of the total variance in the gray‐intensity data. The three channels exhibited negative factor loadings of similar magnitude, indicating that PC1 robustly represents a unified pigmentation index, in which higher values correspond to darker areas and lower values indicate skin lightening. The PCA process is illustrated in Figure 2, while the importance of PC1 is shown in Figure 3.

**FIGURE 2:**
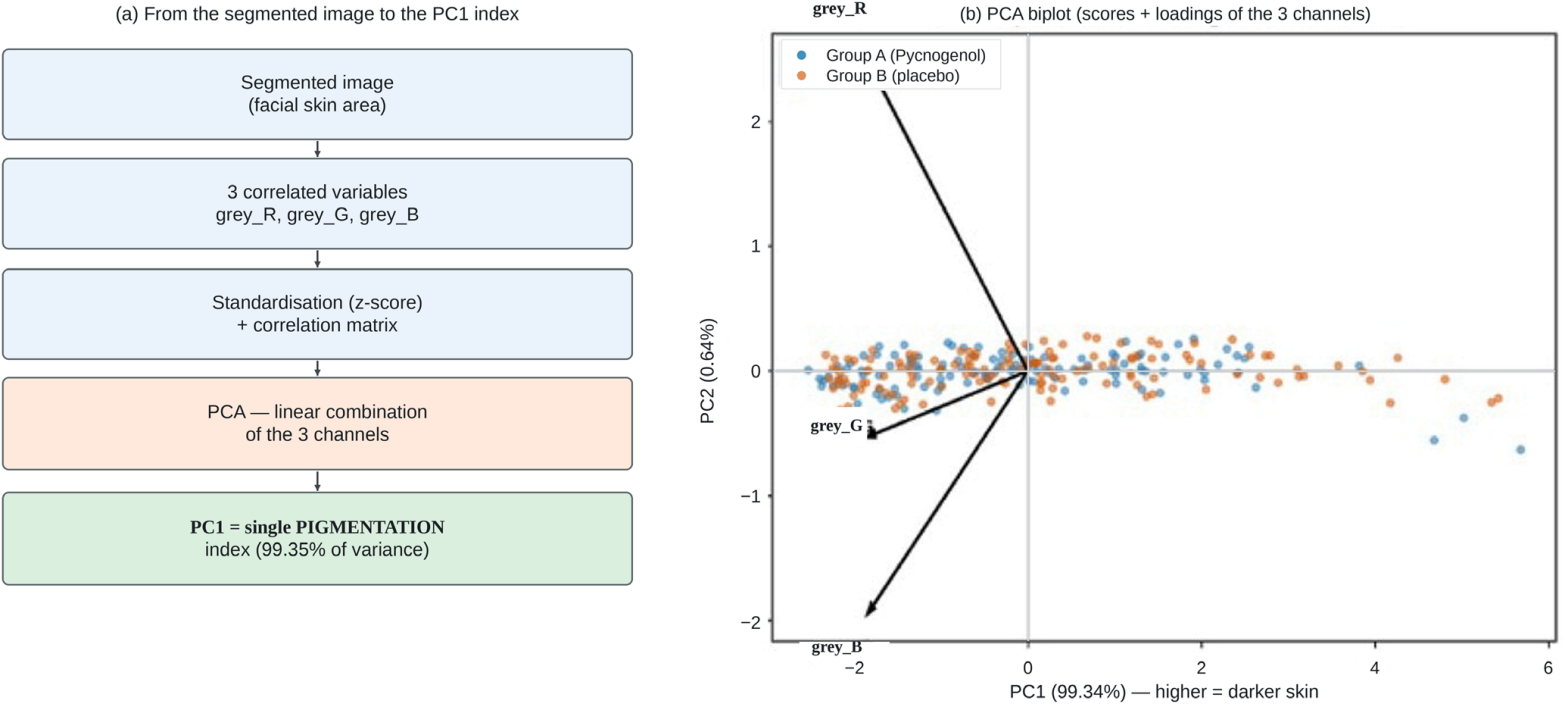
Demonstration of the Principal Component Analysis (PCA) process: (a) workflow from the segmented image to the PC1 index; (b) biplot showing participant scores and loadings of the three gray channels.

**FIGURE 3:**
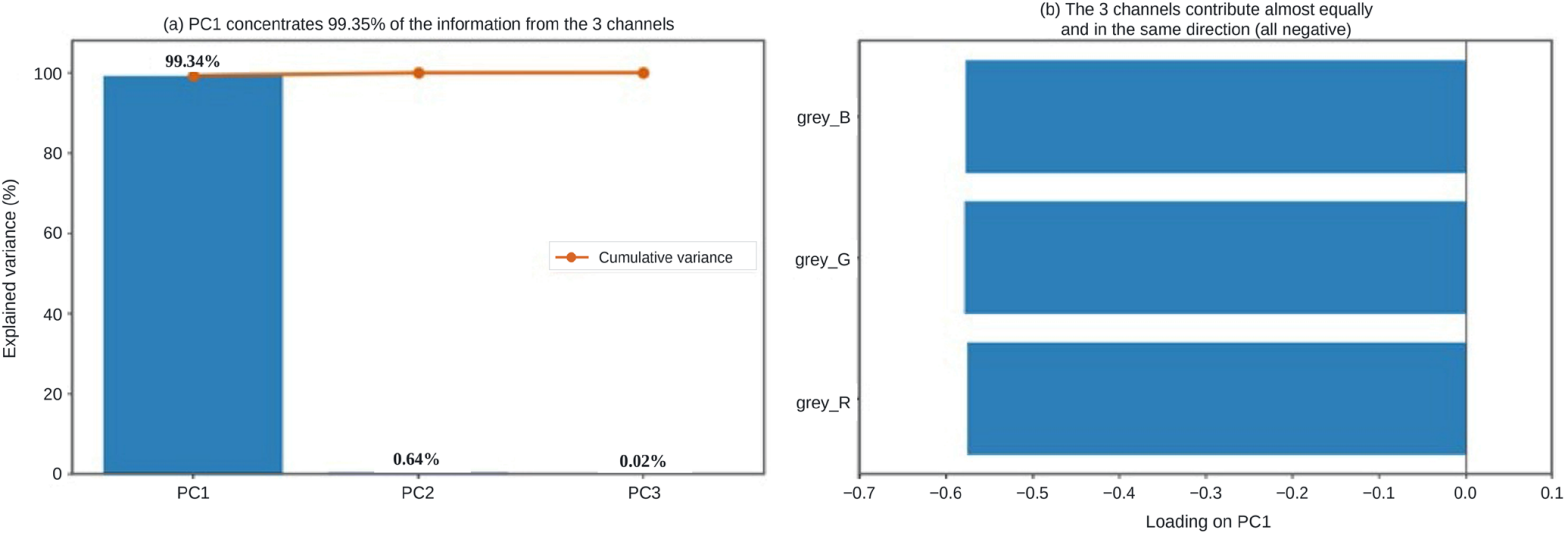
Importance of PC1 as a unified pigmentation index: (a) variance explained by each component (99.35% for PC1); (b) factor loadings of the three channels on PC1.

### 3.3 Linear Mixed‐Effects Model

The LMM identified a significant effect of time on PC1 (*p* = 0.048), indicating that pigmentation changed over time, regardless of group (Table 1). The main effect of group was not statistically significant. Group‐by‐time interaction showed *p* = 0.056, which was considered borderline. This result suggests a trend toward different behavior between groups over time, indicating a possible differential effect of the intervention on melasma lightening.

**TABLE 1:** Analysis of variance (Type III) for fixed effects in the LMM.

Factor	Sum Sq	*F* value	NumDF	DenDF	*p*‐value (Pr(>*F*))
Group	0.730	0.443	1	27.45	0.51
Time	10.114	3.070	2	222.85	**0.04**
Group × time	9.644	2.927	2	222.85	0.05

*Note:* Bold values are statistically significant.

Abbreviations: DenDF, Denominator degrees of freedom; NumDF, Numerator degrees of freedom; Sum Sq, Sum of squares.

The analysis of residuals showed deviations from normality (*p* = 0.003) and heteroscedasticity (*p* = 0.034); however, these violations did not invalidate the model, as LMMs are robust under such conditions. The presence of isolated outliers associated with data from a single patient was also observed; these contributed partially to the distortions but did not compromise the overall data pattern or the clinical interpretation of the results.

### 3.4 Post Hoc Comparisons and Temporal Evolution of Pigmentation

Despise the lack of significance, pairwise comparisons of estimated marginal means revealed clinically relevant trends (Table 2). PC1 was markedly reduced in the intervention group between T_0_ and T_30_ (estimated difference = −0.766; *p* = 0.070), suggesting the greatest lightening effect during the first month. Between T_30_ and T_60_, a slight increase in PC1 was observed (estimated difference = 0.774; *p* = 0.099), possibly due to partial loss or slowing of the initial improvement.

**TABLE 2:** Pairwise comparisons of estimated marginal means for the group‐by‐time interaction.

Contrast	Estimated marginal means	Standard erros	*t*‐ratio	*p*
**A T**_**0**_ **—A T**_**30**_	−0.766	0.279	−2.747	**0.07**
B T_0_—B T_30_	−0.191	0.271	−0.705	0.98
**A T**_**30**_ **—A T**_**60**_	0.774	0.296	+2.610	**0.09**

*Note:* Bold values are statistically significant.

The longitudinal analysis of PC1 (Figure 4) revealed distinct trajectories between the groups. In the placebo group, PC1 increased progressively over the 60‐day period, whereas the treated group showed a transient increase at T30, followed by a return to values close to baseline at T60, ultimately reaching levels below those observed in the placebo group. This divergence between the curves supports the interpretation that the intervention influenced the progression of pigmentation, despite the borderline *p*‐value.

**FIGURE 4:**
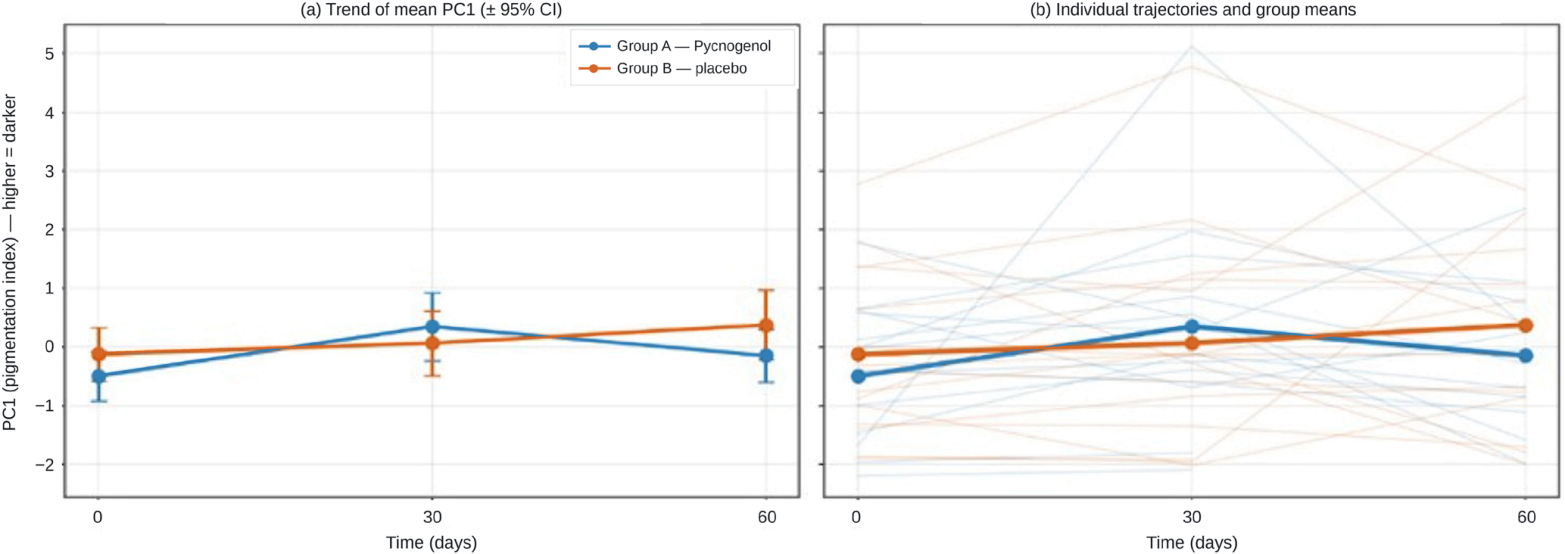
Temporal evolution (trend) of the PC1 pigmentation index by group: (a) mean ±95% CI at T0, T30, and T60; (b) individual trajectories and group means.

None of the within‐group pairwise comparisons reached statistical significance after adjustment, with high *p*‐values observed in the placebo group (*p* > 0.90). These findings reinforce the pattern observed in the LMM, in which the progression of pigmentation differed between the groups over time.

Figure 5 shows the mean PC1 estimate for each group at each time point. At T0, group A (pycnogenol) started with lower values than group B; at T30, both groups showed an increase, which was more pronounced in group A; and at T60, group A returned to lower values, whereas group B remained elevated, resulting in lower estimated pigmentation in the treated group at the end of the study. The wide confidence intervals reflect individual variability.

**FIGURE 5:**
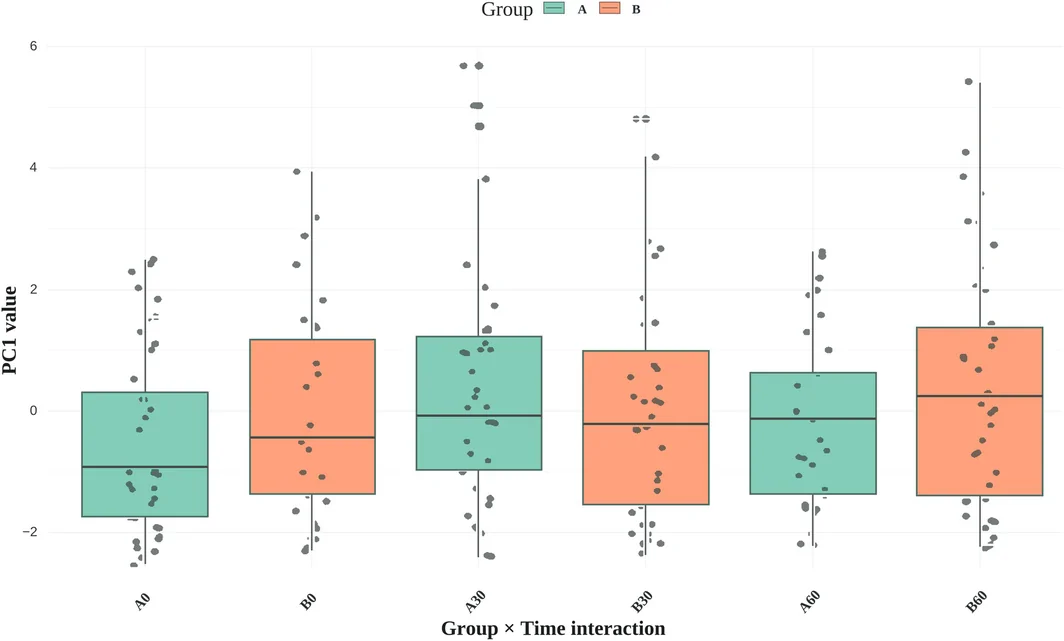
Representation of the pairwise comparisons of estimated marginal means for the Group × Time interaction.

These patterns indicate that the temporal progression differed between the groups, with group B (placebo) tending toward progressive skin darkening and group A (pycnogenol) ending with lower pigmentation. The partial overlap between the groups suggests an absence of pointwise differences, reinforcing that the observed effect is driven by the Group × Time interaction identified in the statistical analyses.

### 3.5 Clinical Interpretation of PC1 Evolution

The longitudinal analysis of PC1 demonstrated that the intervention group had a more pronounced lightening trajectory during the first 30 days, followed by stabilization or slight fluctuation up to 60 days. In contrast, the control group showed curves that remained nearly parallel to the time axis (i.e., no meaningful change during the evaluation period). This divergence between curves indicates that the intervention directly influenced pigmentation changes, despite the borderline *p*‐value.

## 4 Discussion

The results indicated that computational processing of facial images using automated segmentation and gray intensity quantification is sensitive, objective, and reproducible to assess pigmentary changes in individuals with melasma. This approach enabled the dynamic monitoring of subtle variations during treatment and was consistent with recent evidence highlighting the value of digital analysis in pigmentary disorders [6, 8].

The progressive reduction in pigmentation observed in the group treated with pycnogenol, mainly during the first 30 days, is consistent with the pathophysiological behavior of melasma. In general, treatment response may be faster in the early phase, when epidermal pigment predominates and is more accessible to therapeutic interventions [4]. Studies using antioxidant therapies and physical procedures also reported greater clinical response within the first month, followed by a stabilization phase [9].

Understanding the pathophysiology of melasma is important for interpreting the findings of this study. Recent studies indicate that chronic oxidative stress, subclinical dermal inflammation, and melanocyte hyperactivity mediated by reactive oxygen species contributed to the persistence and intensification of pigmentation [2, 10]. In addition, exposure to visible light and near‐infrared radiation may promote melanogenesis and sustained inflammation, contributing to the recurrent nature of the disease [9]. Interventions targeting these pathways may lead to faster responses, particularly in early treatment stages.

The absence of a significant reduction in pigmentation in the control group supports the notion that photoprotection alone may be insufficient to produce marked short‐term lightening. Although sunscreen use may stabilize the condition, it rarely leads to clinically and statistically significant reductions in scores of the modified Melasma Area and Severity Index or digital pigmentation measures [11, 12]. In agreement with these findings, the control group showed a linear trajectory over 60 days, consistent with the natural course of melasma without active treatment.

PCA was sensitive for detecting subtle pigmentary changes. This approach reduces the subjective bias inherent to human evaluation and enables a continuous and comparable quantification over time. Recent studies support the use of PCA and color and texture analysis methods for objective assessments of skin pigmentation, with high reproducibility and correlation with traditional clinical instruments [5, 13].

The improvement observed between T_0_ and T_30_ in the group treated with pycnogenol was followed by a more gradual curve up to T_60_. This pattern is consistent with therapeutic responses described for interventions that modulate melanogenesis and dermal inflammation, in which the initial effects are more pronounced and subsequent changes occur more gradually. Studies using oral antioxidants (e.g., Polypodium leucotomos, vitamin C, and ferulic acid) also reported faster improvements within the first four to six weeks, followed by a slower response phase [14, 15].

Interindividual variability, observed as outliers in some cases, was expected due to the intrinsic heterogeneity of the melasma and differences in phototype, patterns of pigment deposition, and genetic susceptibility. Previous clinical trials using antioxidants, lasers, and chemical peels also reported variability in the responses, indicating individual phenotypes rather than methodological limitations [4, 16].

In addition to treatment‐related findings, this study demonstrated that computational image processing combined with PCA was a viable and robust strategy for objectively quantifying pigmentation in melasma. This approach aligns with current trends in digital tools for clinical monitoring, enabling more precise, automated, and reproducible assessments [8, 13]. The ability to detect early changes, including those not perceptible to the examiner, supports its use as a complementary tool in clinical trials and dermatological practice.

Despite limitations, including a moderate sample size and 60‐day follow‐up period, the findings indicate that computational analysis is robust and accessible to objectively monitor facial pigmentation. Future studies with longer follow‐up, integration of clinical parameters, and use of advanced computer vision techniques may validate these findings and expand their applicability.

## 5 Conclusion

The automated analysis of facial images, based on computational segmentation and gray intensity analysis, demonstrated good consistency in assessing pigmentary changes in individuals with melasma. The PC1‐derived index detected subtle changes over time and distinguished different progression patterns between groups. The intervention group showed a greater reduction in pigmentation during the first 30 days, while the control group remained stable. Although some analyses showed borderline statistical significance for group‐by‐time interaction, the overall pattern supports distinct trajectories between groups, particularly in the early phase.

This methodology reduces subjective bias, ensures reproducibility, and enables quantification of small changes, supporting its use as a complementary approach to traditional methods (e.g., Melasma Area and Severity Index and modified Melasma Area and Severity Index) in clinical practice. Based on these findings, artificial intelligence–assisted image analysis represents a complementary and effective tool for monitoring melasma, improving assessment accuracy, and supporting therapeutic decision‐making in an objective, standardized, and sensitive manner over time.

## Funding

This work was funded by the authors themselves.

## Ethics Statement

Approved by the research ethics committee of the University of Cuiabá (No. 7.042.457). The protocol initiated after ethical approval and written informed consent from all participants.

## Conflicts of Interest

The authors declare no conflicts of interest.

## Data Availability

Data sharing not applicable to this article as no datasets were generated or analysed during the current study.
